# A novel capsule endoscopy for upper and mid-GI tract: the UMGI capsule

**DOI:** 10.1186/s12876-023-02696-5

**Published:** 2023-03-16

**Authors:** Bin Jiang, Yang-Yang Qian, Yuan-Chen Wang, Jun Pan, Xi Jiang, Jia-Hui Zhu, Xiao-Ou Qiu, Wei Zhou, Zhao-Shen Li, Zhuan Liao

**Affiliations:** 1 Department of Gastroenterology, National Clinical Research Center for Digestive Diseases, Changhai Hospital, Shanghai, 200433 China; 2 Department of Gastroenterology, The First Naval Hospital of Southern Theater Command, Zhanjiang, 524005 Guangdong China

**Keywords:** Magnetically controlled capsule endoscopy, Detachable string, Upper and mid gastrointestinal tract, Screening

## Abstract

**Backgrounds and Aims:**

Complete and consecutive observation of the gastrointestinal (GI) tract continues to present challenges for current endoscopy systems. We developed a novel upper and mid gastrointestinal (UMGI) capsule endoscopy using the modified detachable string magnetically controlled capsule endoscopy (DS-MCE) and inspection method and aimed to assess the clinical application.

**Methods:**

Patients were recruited to undergo UMGI capsule endoscopy followed by esophagogastroduodenoscopy. All capsule procedures in the upper gastrointestinal (UGI) tract were conducted under the control of magnet and string. The main outcome was technical success, and the secondary outcomes included visualization of the UMGI tract, examination time, diagnostic yield, compliance, and safety evaluation.

**Results:**

Thirty patients were enrolled and all UMGI capsule procedures realized repeated observation of the esophagus and duodenum with detection rates of 100.0%, 80.0%, and 86.7% of Z-line, duodenal papilla, and reverse side of pylorus, respectively. String detachment was succeeded in 29 patients (96.7%) and the complete examination rate of UMGI tract was 95.45% (21/22). All UMGI capsule procedures were well tolerated with low discomfort score, and had a good diagnostic yield with per-lesion sensitivity of 96.2% in UGI diseases. No adverse events occurred.

**Conclusions:**

This new capsule endoscopy system provides an alternative screening modality for the UMGI tract, and might be indicated in cases of suspected upper and small bowel GI bleeding.

*Trial registration* DS-MCE-UGI and SB, NCT04329468. Registered 27 March 2020, https://clinicaltrials.gov/ct2/results?cond=&term=NCT04329468.

## Background

Patients with gastrointestinal (GI) bleeding are recommended to receive esophagogastroduodenoscopy (EGD), colonoscopy and capsule endoscopy [1, 2], and of them the incidence of upper and mid gastrointestinal (UMGI) lesions in patients taking antithrombotic or non-steroidal anti-inflammatory drugs can be up to about 80% [3, 4]. It may delay diagnosis and treatment due to the step-by-step strategy. The development of magnetically controlled capsule endoscopy (MCE) with an excellent ability for diagnosis of gastric diseases proved to be noninvasive and painless and has shown great potential in saving screening steps, time, and money compared with the standard strategy [5, 6]. Moreover, it is confirmed recently that capsule endoscopy can reduce the number of invasive endoscopy procedures, unnecessary personal protective equipment, and exposure to SARS-CoV-2 in patients with acute GI bleeding during the COVID-19 pandemic [7], and can also detect and monitor effectively gastric and small intestinal injuries induced by antiplatelet therapy [8].

However, complete and consecutive observation of the GI tract continues to present challenges under capsule endoscopy [9]. For example, insufficient visualization of the esophagus and duodenum, which is indicated by infrequent identification of Z-line and duodenal papilla, occurs due to rapid transit of capsule without effective control and technical limitations in frame rate and view angle [10]. In addition, consecutive examination for the UMGI tract is essential for earlier diagnosis of GI injuries.

Ching HL [11] reported that PillCam upper gastrointestinal (UGI) capsule using a nurse-led protocol achieved esophagogastric examination, but a third of patients could not complete duodenal inspection due to limited battery. Lien GS [12] developed a novel magnetic-assisted capsule endoscopy consisted of a cable capsule and hand-held magnet, proved satisfactory visualization in the UGI tract, could not further inspect small bowel due to the limitation of cable.

In our previous studies [13, 14], second-generation MCE and detachable string MCE (DS-MCE) have been confirmed to perform better UGI tract visualization, especially in the esophagus and stomach. Here we developed a novel UMGI capsule endoscopy system using modified DS-MCE and inspection method, and conducted a pilot study to evaluate its safety and feasibility for complete and consecutive UMGI examination.

## Methods

### Study design

This pilot study was a prospective, single-centered, blinded self-controlled study. The study protocol was approved by the Institutional Review Board of Changhai Hospital (Shanghai, China) and registered at ClinicalTrials.gov. with registration number NCT04329468. All enrolled subjects signed the informed consents according to the Helsinki Declaration.

### Study patients

Patients aged from 18 to 80 years old and willing to undergo both UMGI capsule and EGD examination with or without gastrointestinal complaints were prospectively recruited in Changhai Hospital from March to August 2020, in which the asymptomatic subjects were healthy volunteers. Examination of UGI tract with/without small bowel were confirmed according to their clinical manifestations and requirements. Basic characteristics, gastrointestinal complaints, and *Helicobacter pylori* infection status were recorded. Patients with the following criteria were excluded: (1) those with suspected or known gastrointestinal stenosis, obstruction, fistula, or other risk factors for capsule retention; (2) metallic or electronic medical devices implanted; (3) those were pregnant or suspected of pregnancy; (4) unable to cooperate with the examination such as psychotics or in poor general condition of hemodynamic instability; and (5) those with any other contraindications to MCE or EGD procedure determined by endoscopists.

### Study intervention

Each participant underwent UMGI capsule endoscopy followed by EGD within one week, and all endoscopy examinations were conducted before treatment of GI diseases. UMGI capsule was carried out by an endoscopist with an experience of more than 1000 cases of MCE procedures and a physician assisting in the control of detachable string. Standardized EGD examination with xylocaine spray for pharyngeal local anesthesia was performed by another well-trained endoscopist, who was blinded to the capsule findings. The final diagnosis was made by combining the results of both modalities. During these procedures, a designated researcher collected the relative data about all evaluation parameters.

### UMGI capsule procedure

UMGI capsule endoscopy system (Ankon Technologies Co. Ltd, Shanghai, China) consists of a detachable string attached to the capsule and an upgraded MCE [13] with an adaptive frame rate of 8 frames per second, image resolution of 720 × 720 pixels, field of view of 150 degrees, and battery duration of more than 12 h. This hollow and thin string is composed of sterile and transparent latex for single use, 120 cm in length with a caudal suction cap. Capsule endoscopy is partially enclosed within the cap and can be detached from the string by injecting air with a sterile syringe [14].

**Fig. 1:**
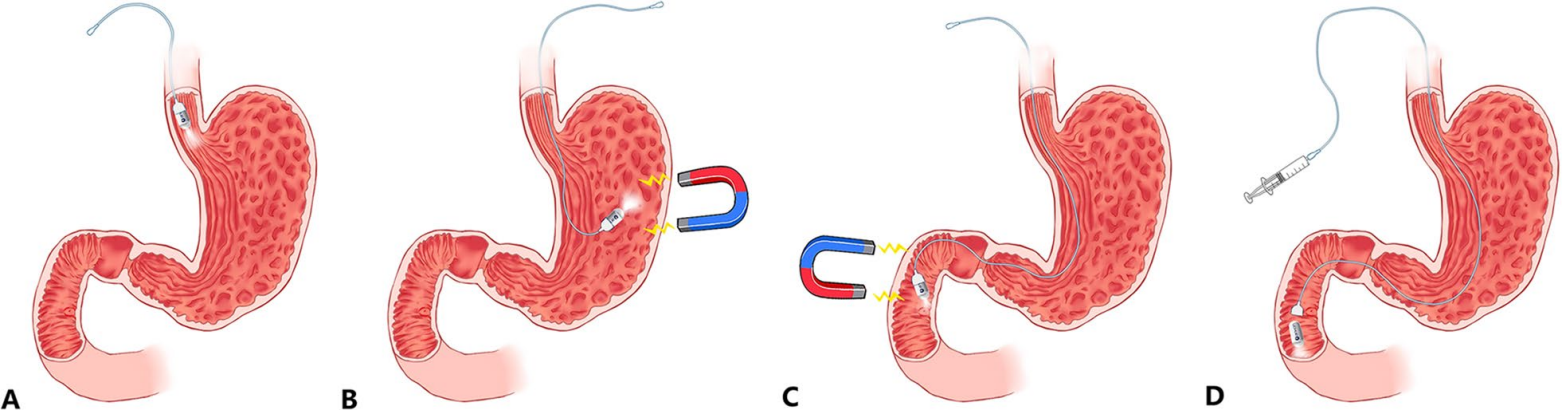
Examination strategy of the UMGI capsule endoscopy. Esophageal observation under string control (**A**); Capsule with string was controlled by external magnet to inspect the whole stomach (**B**); Capsule was allowed to repeatedly view duodenum including duodenal papilla and the reverse side of pylorus under the combined control of magnet and string (**C**); Capsule was separated from string and proceed with small-bowel examination (**D**). Source: This Fig. 1 was originally designed and made by our research team, which was permitted by all authors before the submission and up to now not published anywhere

After a standardized preparation regimen [15] (Bowel preparation: patients received 2 L polyethylene glycol 5 h before examination. Gastric preparation: patients ingested 400 mg simethicone suspension dissolved in 100 mL water 40 min before examination and drank 1000 mL of water 10 min before examination.), patients swallowed the capsule in left lateral position, allowing the capsule to be slowly pulled up and down in esophagus under string control for repeated observation of targeted areas under real-time viewing. Water ingestion would be repeated for adequate visualization of esophageal mucosa. After completing esophageal examination, the capsule entered the stomach and was lifted, rotated, advanced or returned to orderly observe gastric fundus, cardia, body, angulus, antrum and pylorus, controlled by external magnet robot [15]. When stomach examination was finished, magnetic steering was used to help the capsule pass through pylorus [16], and the string was tension-free during the process. After entering duodenum, the capsule was allowed to gradually travel down as far as the end part of duodenum and then be slowly pulled up by the string for complete viewing of duodenal mucosa. Detection of duodenal papilla and the reverse side of pylorus in duodenal bulb was tried within a maximum of three-time repeated viewing under the combined control of magnet and string, in which “360-degree automatic scanning” mode was used to help rotate the capsule. If mucus, bubbles or insufficient distension impeded the observation, a small amount of water ingestion and position changes in supine or right-lateral decubitus might be helpful and were allowed as needed. If discomfort caused by the string occurred, manipulator would slow down or stop the movement of the string and let the patients regulate their breath, and wait for the next right time unless the patients decided to quit the trial. Once completing duodenal examination, the capsule was separated from the string and proceeded with small-bowel examination (if needed) without external magnet force according to the standardized protocol (Fig. 1).

### Study outcomes

The primary study outcome was technical success of UMGI capsule endoscopy, defined as successful repeated observation of esophagus, stomach, and duodenum and detachment of capsule from string after duodenal examination. Secondary outcomes included the visualization of UMGI tract, examination time, diagnostic yield, discomfort and adverse events associated with the procedure.

Visualization of esophagus was indicated by the detection of Z-line defined as at least one image of Z-line was obtained and circumferential viewing of Z-line (one quadrant; at least two quadrants; at least three quadrants; and all four quadrants) [14]. Visualization of gastric mucosa in each anatomical landmark was evaluated as good, moderate, and poor defined as 90–100%, 70–90% and < 70% of mucosa could be observed, respectively [5]. Visualization of duodenum was indicated by the detection of duodenal papilla and the reverse side of pylorus in duodenal bulb, and was graded as full, more than half and less than half being viewed [17]. Discomforts such as nausea, vomiting or cough were scored on a scale from 0 to 3 (0, none; 1, mild; 2, moderate; and 3, severe), and overall discomfort was graded using a scale of 0 to 10 (0, none; 10, severe) [14].

### Statistical analysis

As a pilot study to evaluate the clinical utility of the UMGI capsule, sample size was not calculated. Quantitative data were presented as mean ± standard deviation (SD) or median and interquartile range (IQR), where appropriate, and categorical data were described as frequency (percentage). Statistical analyses were performed using SPSS software version 25.0 (IBM Corp, Armonk, NY, USA).

## Results

### Patient characteristics

A total of 30 subjects (mean age 31 years, range 19–65 years; male 66.7%) were enrolled and analyzed, which consisted of 12 asymptomatic volunteers and 18 patients with gastrointestinal complaints including abdominal pain, distention, acid reflux and nausea or vomiting. 4 subjects had a history of *Helicobacter pylori* infection, 8 subjects received UGI tract examination and 22 participants received UGI tract and small bowel examination. The mean interval between UMGI capsule and EGD procedure is 3.63 days (range 1–7 days).

### Feasibility and safety analyses

Successful repeated observation in esophagus, stomach and duodenum was achieved in all patients, with detachment of capsule from string failed in one patient, and technical success rate was 96.7% (29/30). Among 22 patients demanding small bowel inspection, 21 (95.5%) achieved complete examination with cecal images and the other one failed caused by ileal ulcer with stenosis. No adverse events including anaphylaxis and capsule retention were reported.

### Visualization of esophagus, stomach and duodenum

**Fig. 2:**
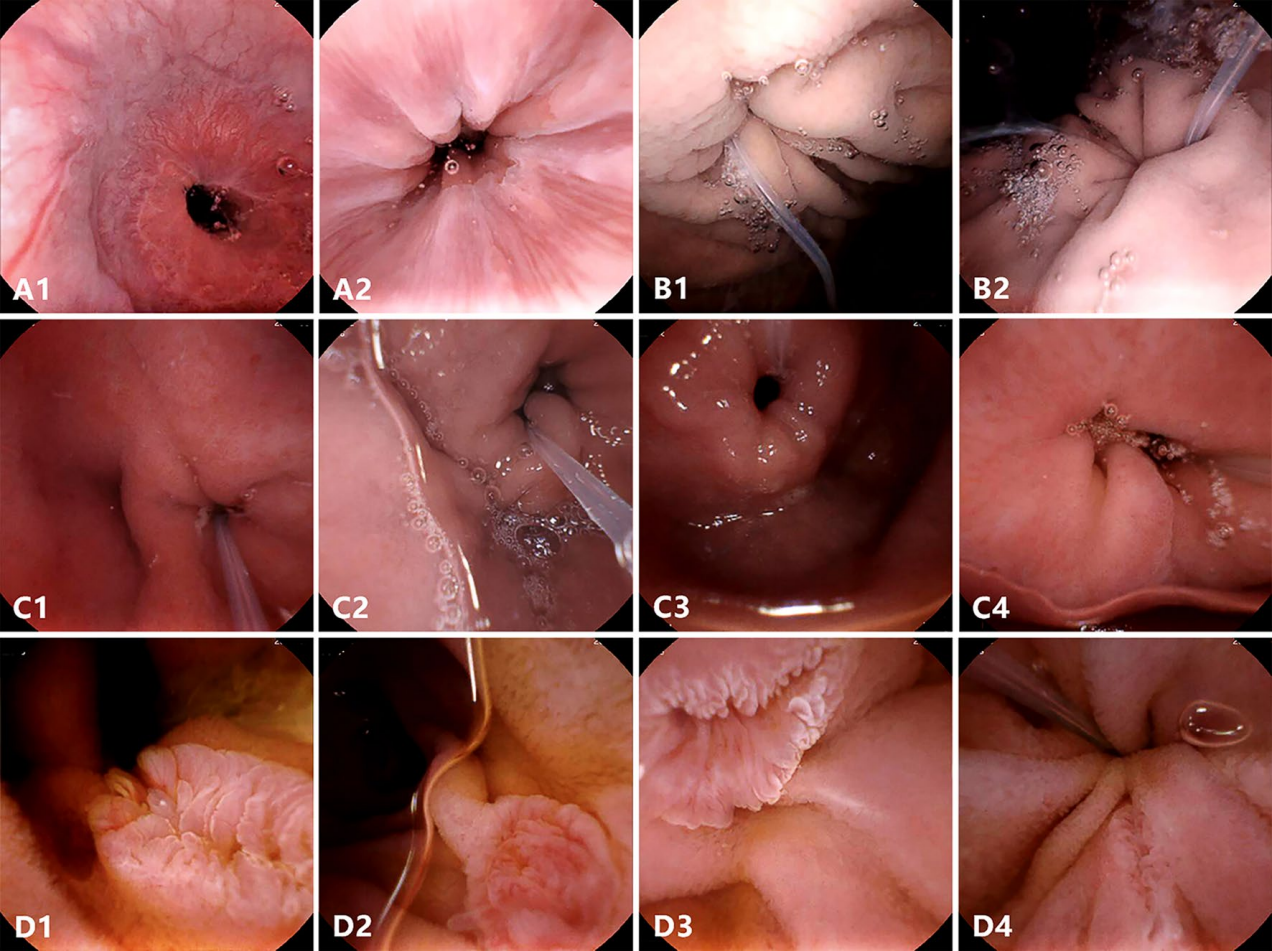
Representative images of UGI anatomical landmarks under the UMGI capsule endoscopy. Complete viewing of Z-line (**A1**, **A2**); The close-up image of Cardia (**B1**, **B2**); The reverse side of pylorus in duodenal bulb (**C1-4**); The major duodenal papilla with different kind of shapes (**D1-4**)

UMGI capsule endoscopy provided satisfactory visualization of UGI tract mucosa (Table 1 and Fig. 2). In gastroesophageal junction, Z-line was detected in 30 (100%) patients, and circumferential visualization with all four quadrants was achieved in 20 (66.7%) patients. In stomach, visualization of gastric mucosa at main anatomical landmarks (fundus, cardia, body, angulus, antrum, and pylorus) were all assessed as good (≥ 90%) in all patients, and close viewing of gastric cardia and fundus were easily achieved in 24 (80.0%) patients. In duodenal bulb, the UMGI capsule detected the reverse side of pylorus in 26 (86.7%) patients, obtaining a full view in 20 (66.7%) patients and more than half view in 25 (83.3%) patients. The detection rate of duodenal papilla was 80.0% (24/30), presenting different kinds of appearance as villus, granule, fissure and longitudinal aperture. Full view of duodenal papilla was captured in 17 (56.7%) patients and more than half view was captured in 24 (80.0%) patients.

**Table 1:** Visualization of UGI tract under UMGI capsule endoscopy in 30 patients

Characteristics	Results, n (%)
Detection rate of Z-line	30 (100)
*Circumferential viewing of Z-line*	
4 quadrants	20 (66.7)
≥ 3 quadrants	25 (83.3)
≥ 2 quadrants	28 (93.3)
≥ 1 quadrant	30 (100)
*Visualization of gastric mucosa*	
Fundus	30 (100)
Cardia	30 (100)
Body	30 (100)
Angulus	30 (100)
Antrum	30 (100)
Pylorus	30 (100)
Detection of the reverse side of Pylorus	26 (86.7)
*Visualizing level of the reverse side of Pylorus*	
Full	20 (66.7)
More than half	5 (83.3)
Less than half	1 (3.3)
Detection of duodenal papilla	24 (80.0)
*Visualizing level of duodenal papilla*	
Full	17 (56.7)
More than half	7 (23.3)
Less than half	0 (0)

### Discomfort assessment

Most patients felt no or mild discomfort during UMGI capsule endoscopy procedure (Table 2). Mean discomfort scores when swallowing capsule, capsule being pulled up and down in esophagus and duodenum, string being in throat during gastric examination, and string being pulled out were 0.60 (range 0–3), 0.63 (range 0–2), 0.17 (range 0–1), 1.00 (range 0–3), and 0.47 (range 0–3), respectively. The mean overall discomfort score compared with EGD was 1.37 (range 0–3).

**Table 2:** Discomfort scores associated with the string during UMGI capsule procedure

Score	0, n (%)	1, n (%)	2, n (%)	3, n (%)	Mean score (range)
Swallowing capsule	16 (53.3)	11 (36.7)	2 (6.7)	1 (3.3)	0.60 (0–3)
Pulling capsule up and down in esophagus	14 (46.7)	13 (43.3)	3 (10.0)	0 (0)	0.63 (0–2)
String in throat during gastric examination	25 (83.3)	5 (16.7)	0 (0)	0 (0)	0.17 (0–1)
Pulling capsule up and down in duodenum	8 (26.7)	16 (53.3)	4 (13.3)	2 (6.7)	1.00 (0–3)
Pulling out the string	19 (63.3)	9 (30.0)	1 (3.3)	1 (3.3)	0.47 (0–3)
Overall discomfort	5 (16.7)	13 (43.3)	8 (26.7)	4 (13.3)	1.37 (0–3)

### Examination time

The median capsule swallowing time and esophageal examination time were 9.5 s (IQR, 6.0–19.25 s) and 2.42 min (IQR, 1.95–3.00 min, range 1.47–7.88 min), respectively. The mean gastric examination time and duodenal examination time were 6.24 ± 0.98 min and 12.06 ± 4.71 min (range 4.48–23.95 min), respectively. The median pyloric transit time and mean small bowel transit time were 44.50 min (IQR, 11.11–82.96 min) and 297.04 ± 68.86 min, respectively.

### Diagnostic performance in UMGI tract

**Fig. 3:**
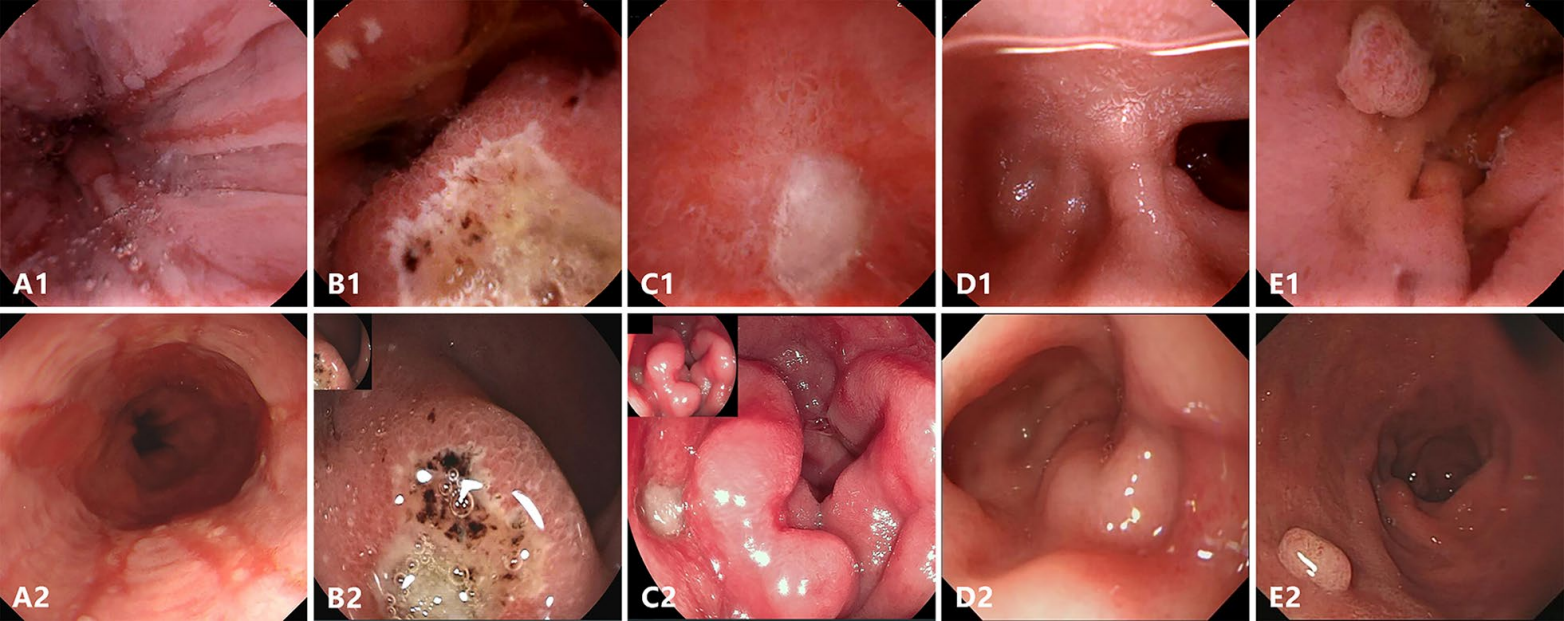
Representative UGI lesions detected by both the UMGI capsule endoscopy (upper panel) and EGD (lower panel). Reflux esophagitis (**A**); Ulcer in the anterior (**B**) and posterior (**C**) wall of duodenal bulb; Diverticulum in duodenal bulb (**D**); Polyp in duodenum (**E**)

**Fig. 4:**
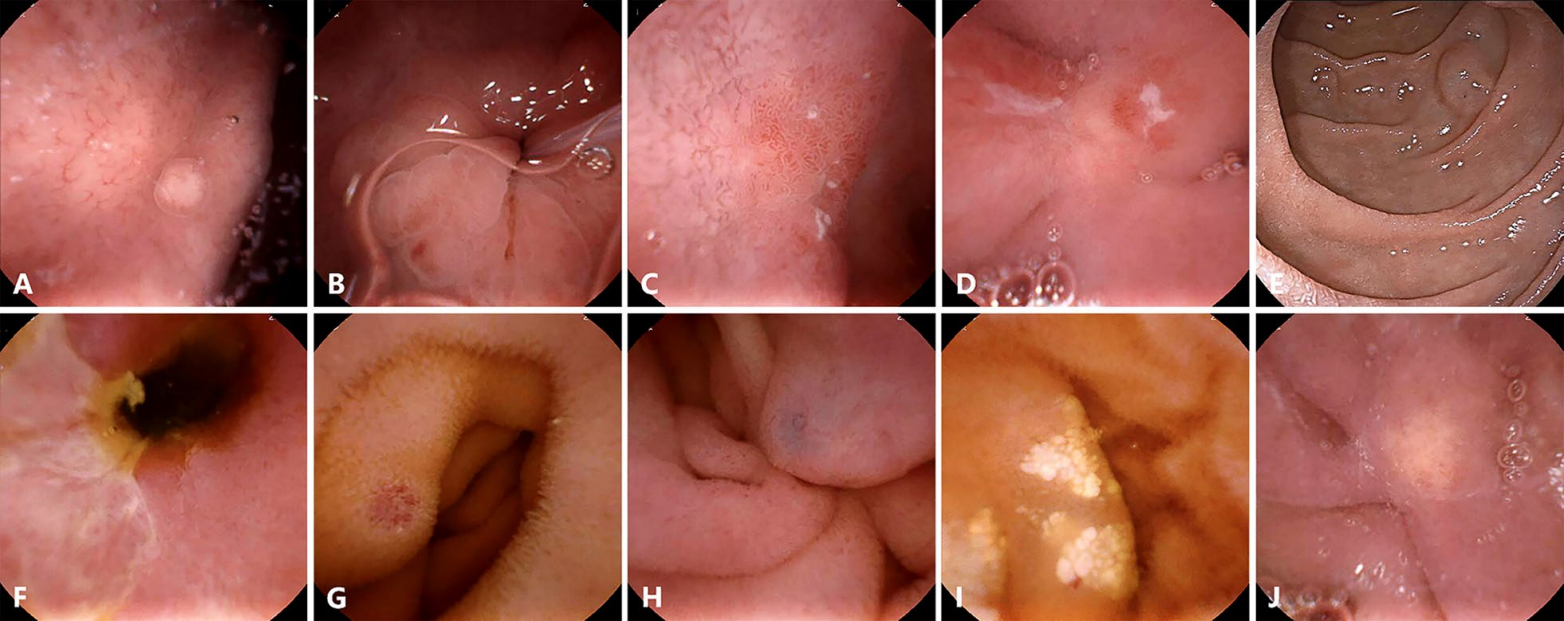
UGI lesions missed under the UMGI capsule or EGD and small-bowel lesions detected by the UMGI capsule. Lesions missed under EGD: Gastric polyp (**A**); Erosion (**B**, **C**) and Ulcer **D** in duodenal bulb; Lesions missed under the UMGI capsule endoscopy: Submucosal mass in the descending part of duodenum (**E**); Lesions in small bowel: Ulcer (**F**); Angiodysplasia (**G**, **H**); Lymphangiectasia (**I**); Xanthoma (J)

As described in Table 3, Figs. 3 and 4, a total of 32 lesions in the UGI tract were detected, 27 of which were diagnosed by both methods. With EGD as the gold standard, the sensitivity for detecting all UGI lesions under capsule was 96.2% (25/26) and 100% (17/17) in per-lesion and per-patient analysis respectively. EGD detected one additional submucosal mass in the descending part of duodenum, and UMGI capsule endoscopy found four lesions missed by EGD, including one polyp in gastric body, one ulcer and two erosions in duodenal bulb. Of note, UMGI capsule endoscopy also detected eleven abnormal findings in small bowel, including one ulcer, two erosions, four lymphangiectasia, one xanthoma and three angiodysplasia. Erosion or ulcer in UMGI tract were detected in 2 (16.7%) asymptomatic volunteers and 11 (61.1%) patients with gastrointestinal complaints.

**Table 3:** Classification of lesions diagnosed by UMGI capsule endoscopy and EGD

Lesions	Both examination, n	Capsules only, n	EGD only, n
*Esophagus*			
Reflux esophagitis	3	0	0
*Stomach*			
Atrophic gastritis	3	0	0
Erosion	9	0	0
Polyp	1	1	0
Ulcer	2	0	0
*Duodenum*			
Erosion	3	2	0
Polyp	1	0	0
Ulcer	4	1	0
Diverticulum	1	0	0
Submucosal mass	0	0	1
*Small bowel*			
Erosion	–	2	–
Ulcer	–	1	–
Angiodysplasia	–	3	–
Xanthoma	–	1	–
Lymphangiectasia	–	4	–

## Discussion

This is the first study that assessed the feasibility and safety of a novel UMGI capsule endoscopy. Our results confirmed that the UMGI capsule could successfully repeat the examination and had sufficient observation of UGI tract in all patients, and realized complete and consecutive UMGI examination within one capsule. This study optimized clinical application of MCE and further benefited patients who require whole UMGI tract examination, including those with definite or suspected GI bleeding, and taking antithrombotic or non-steroidal anti-inflammatory drugs with gastrointestinal complaints [2].

Our study highlighted the role of UMGI capsule in duodenal visualization. The major duodenal papilla presented a good surrogate maker of lesions in proximal small bowel for capsule examination, its identification was still unsatisfactory although improvement of technical parameters might promote the visualization [17–19]. In this study, we achieved a detection rate of 80%, implying the importance of accurate control for transit time and capsule orientation in tubular structure, and making the performance of the capsule close to EGD, under which the detection rate of duodenal papilla was from 70 to 90% [20]. The reverse side of pylorus in the GI junction, regarded as the blind spot of forward-viewing EGD and where lesions also might exist [21], could be viewed by capsule endoscopy when it passed through the duodenal bulb with tail-first orientation. Nevertheless, single-camera capsules randomly passed through the pylorus tail-first in a minority of patients because intestinal peristalsis tended to drive the lighter head containing camera first [22]. Under the control of magnet and string in our study, capsule was successfully guided to reverse its direction to get retrograde view of pylorus in 86.7% of patients, further extending the examination field.

UMGI capsule also presented a satisfactory visualization in esophagus and stomach with controllable movement and real-time viewing, merging the advantages of string capsule and MCE. The detection rate of Z-line was 100% with a complete circumferential viewing of 66.7%, similar to that of string capsule [23, 24] and seemed superior to that of esophageal capsule endoscopy without string [25]. Moreover, esophageal examination time was shortened from 5.08–6.20 min [14, 23] to 2.42 min in this study, probably on account of increased operation experience, improved frame rate and view angle. UMGI capsule also further optimized the observation of gastric cardia and fundus by making the close shot easily located by the string, without influence on inspection integrity and examination time. A study using DS-MCE showed that distal gastric observation was limited by the pull of string with 80 cm in length [24], and our study used a longer string of 120 cm and achieved flexible capsule movement in whole stomach and the proximal small bowel.

This study confirmed the diagnostic ability of UMGI capsule in esophageal diseases with a high consistency with EGD, showing a promising utility in detecting gastroesophageal varices in patients with liver cirrhosis [26, 27]. For gastric diseases, the UMGI capsule also showed excellent performance based on previous MCE. Diagnosis of duodenal diseases in comparison with EGD was rarely explored so far, and significant pathologies may be missed under capsule such as duodenal ulcers, polyps and adenocarcinoma [21]. Although the diagnostic efficacy of the UMGI capsule is acceptable, being unable to take biopsy really limits the confirmation of *Helicobacter pylori* infection and precancerous lesions, and unable to wash gastric mucosa during inspection require a good gastric preparation and multiple body position changes. Our results showed an excellent capability of UMGI capsule in detecting duodenal lesions, and more mucosal lesions were detected such as erosion and ulcer, 2 of which were located in the reverse side of pylorus. Of note, one submucosal mass suspected as a cyst in the second part of duodenum was missed under capsule, possibly hindered by the folds and loop angulations despite of water ingestion and position changes, indicating that sufficient distention of intestine still needs investigation.

The detachable string helped effective control of the capsule, contributed to a successful consecutive inspection of the UMGI tract, and avoided the discomfort caused by pulling the capsule out [25]. The major discomfort was caused by the irritation of throat while pulling up and down the string, and was evaluated as none and mild in most patients, similar to those in previous studies [14, 24]. UMGI capsule resulted in high acceptability with little discomfort compared with EGD without sedation. Although the EGD procedure with sedation and MCE without string are more comfortable, adverse events associated with sedation may occur during EGD and incomplete observation of UGI tract may occur under MCE. Failure of the detachment, reported in previous researches [23], happened in one patient after duodenal examination, possibly due to an occasional twist of string impeding the air injection. As this malfunction was also reported in esophagus, confirmation of successful detachment before ingestion may help avoid the failure.

There are limitations in this trial. First, excellent diagnostic ability of UMGI capsule endoscopy with a small sample size in the pilot study merits validation in large-scale trials. Second, the longer operation time for the UGI tract under UMGI capsule endoscopy than that of EGD, especially during pyloric transit of the capsule, promotes further innovations.

## Conclusion

Our study provided a safe and feasible modality for complete and consecutive visualization of UMGI tract with high tolerance and diagnostic yield, and UMGI capsule might be indicated in cases of suspected upper and small bowel GI bleeding, possibly associated with or due to the use of anticoagulant/antiplatelet or non-steroidal anti-inflammatory therapy, especially in those who are unwilling and unable to undergo traditional endoscopy. The study provided a basis for innovation of a real “mouth to anus” screening tool in the future.

## Data Availability

The datasets generated and analyzed during the current study are not publicly available due to health privacy concerns, but are available from the corresponding author on reasonable request.

## Abbreviations

DS-MCE: Detachable string magnetically controlled capsule endoscopy
UGI: Upper gastrointestinal
UMGI: Upper and mid gastrointestinal
EGD: Esophagogastroduodenoscopy
